# The effects of therapeutic hip exercise with abdominal core activation on recruitment of the hip muscles

**DOI:** 10.1186/s12891-017-1674-2

**Published:** 2017-07-21

**Authors:** Mandy KY Chan, Ka Wai Chow, Alfred YS Lai, Noble KC Mak, Jason CH Sze, Sharon MH Tsang

**Affiliations:** Department of Rehabilitation Sciences, The Hong Kong Polytechnic University, Hunghom, Hong Kong SAR, China

**Keywords:** Hip muscles, Electromyography, Abdominal core activation

## Abstract

**Background:**

Core stabilization has been utilized for rehabilitation and prevention of lower limb musculoskeletal injuries. Previous studies showed that activation of the abdominal core muscles enhanced the hip muscle activity in hip extension and abduction exercises. However, the lack of the direct measurement and quantification of the activation level of the abdominal core muscles during the execution of the hip exercises affect the level of evidence to substantiate the proposed application of core exercises to promote training and rehabilitation outcome of the hip region. The aim of the present study was to examine the effects of abdominal core activation, which is monitored directly by surface electromyography (EMG), on hip muscle activation while performing different hip exercises, and to explore whether participant characteristics such as gender, physical activity level and contractile properties of muscles, which is assessed by tensiomyography (TMG), have confounding effect to the activation of hip muscles in enhanced core condition.

**Methods:**

Surface EMG of bilateral internal obliques (IO), upper gluteus maximus (UGMax), lower gluteus maximus (LGMax), gluteus medius (GMed) and biceps femoris (BF) of dominant leg was recorded in 20 young healthy subjects while performing 3 hip exercises: Clam, side-lying hip abduction (HABD), and prone hip extension (PHE) in 2 conditions: natural core activation (NC) and enhanced core activation (CO). EMG signals normalized to percentage of maximal voluntary isometric contraction (%MVIC) were compared between two core conditions with the threshold of the enhanced abdominal core condition defined as >20%MVIC of IO.

**Results:**

Enhanced abdominal core activation has significantly promoted the activation level of GMed in all phases of clam exercise (*P* < 0.05), and UGMax in all phases of PHE exercise (*P* < 0.05), LGMax in eccentric phases of all 3 exercises (*P* < 0.05), and BF in all phases of all 3 exercises except the eccentric phase of PHE exercise (*P* < 0.05). The %MVIC of UGMax was significantly higher than that of LGMax in all phases of clam and HABD exercises under both CO and NC conditions (*P* < 0.001) while the %MVIC of LGMax was significantly higher than UGMax in concentric phase of PHE exercise under NC condition (*P* = 0.003). Gender, physical activity level and TMG parameters were not major covariates to activation of hip muscles under enhanced core condition.

**Conclusions:**

Abdominal core activation enhances the hip muscles recruitment in Clam, HABD and PHE exercises, and this enhancement is correlated with higher physical activity and stiffer hip muscle. Our results suggest the potential application of abdominal core activation for lower limb rehabilitation since the increased activation of target hip muscles may enhance the therapeutic effects of hip strengthening exercises.

## Background

Over past two decades, core stabilization has been popular in rehabilitation and sports training program to prevent musculoskeletal injuries and enhance performances [1, 2]. Both the lumbar spine and pelvis can be stabilized by the passive and active subsystems of the neuro-musculo-skeletal system [3]. The integrity and interaction of the bony structures and soft tissues of the vertebral column contribute to the role and function of the passive subsystem. Muscles which embrace the abdominal wall, the intersegmental muscles and the para-spinal muscles form the active subsystem. By promoting the activation of the abdominal core muscles, the composite function of the active subsystem in promoting the spinal stability could therefore be enhanced.

Previous studies have used pressure biofeedback unit (PBU) to indirectly monitor the magnitude of abdominal core activation and examined its effect on hip muscles activity in the isometric phases of side-lying hip abduction and prone hip extension exercises [4–6]. It remains questionable if this method warrants the validity of the abdominal core recruitment because the value shown in the PBU is primarily from the changes in pressure that the body segment exerts upon the transducer. Factors like body weight and displacement of the center of mass during exercises would have therefore limited the comparisons between participants and exercises [7]. Furthermore, the effect of abdominal core activation was only examined during the isometric phase of exercises. In studies conducted by Chance-Larsen et al. [4] and Oh et al. [5], the activity of gluteus maximus (GMax) was assessed as a single unit in which the functional differences of the subdivisions of GMax were not considered [8].

Therefore, the present study was designed to monitor the abdominal core activation directly over the bilateral internal oblique muscles (IO) using the surface EMG method. The effect of abdominal core activation was also examined more comprehensively with the inclusion of analysis of muscle activity of the subdivisions of GMax and additional exercises and phases. This study aimed to examine the effects of abdominal core activation on hip muscles activity during therapeutic hip exercises with regards to the concentric, isometric and eccentric phases. Furthermore, selected factors which included the gender and physical activity level of the participants and the contractile properties of hip muscle assessed by the tensiomyography (TMG) were also examined for the possible confounding effects these factors may have on the activation of the hip muscles during the therapeutic hip exercises.

## Methods

### Study design

This was a cross-sectional study. The independent variables were two condition of abdominal core activation: 1) natural core activation (NCA) and 2) enhanced core activation (ECA). The ECA condition was defined as at least 20% of the normalized percentage of maximum voluntary isometric contraction (%MVIC) of the contralateral and ipsilateral internal obliques (CIO & IIO) of the dominant leg (the side used to kick a ball). A target of 20% of MVIC of the IO was adopted in this study based on the level of abdominal core activation which could offer optimal spinal stability as suggested in previous research [9, 10]. The dependent variables were the activity of gluteus medius (GMed), upper part of gluteus maximus (UGMax), lower part of gluteus maximus (LGMax) and biceps femoris (BF) of the dominant leg measured by the surface electromyography (EMG).

### Participants

Twenty healthy participants (10 F and 10 M) were recruited from the Hong Kong Polytechnic University using convenient sampling. Participants with any musculoskeletal or neurological disorder over their lower back or lower limbs were excluded. Explanation of objectives, procedures, benefits and potential risks of the study was given before participants signed an informed consent approved by the Ethics Committee of the Hong Kong Polytechnic University. Demographic data, including the physical activity level, measured by International Physical Activity Questionnaire Short Form (IPAQ-SF) [11], and the contractile properties of GMax (maximal radial displacement [Dm] and contraction time [Tc]), measured by TMG system (TMG S1 system, TMG-BMC Ltd., Slovenia), was shown in Table 1. The TMG measurement enables the assessment of the muscle mechanical response towards an electrical stimulus using an non-invasive approach [12]. Both the Dm and Tc values provide an objective and reliable measure of the contractile property of the muscle. Due to the inaccessibility of the deep gluteal muscles, only the GMax was measured in this study.

**Table 1:** Characteristics of participants with mean (SD)

Age (years)	21.10 (1.70)		
Height (cm)	166.75 (7.90)		
Weight (kg)	58.10 (9.20)		
Dominant leg (n)	Left = 0	Right = 20	
Physical activity level (n)	Low = 0	Moderate = 10	Vigorous = 10
Contractile properties			
Maximal radial displacement, Dm (mm)	9.72 (3.15)		
Contraction time, Tc (ms)	42.42 (5.32)		

### Experimental procedures and measurements

For monitoring of the muscle activity of the transverse abdominal wall, EMG activity of the transverse fibres of the internal obliques and the underlying transverse abdominis, which was named as IO in this study, was measured using the surface EMG methods [9, 13, 14]. Before placing EMG electrodes, skin preparation including hair removal, light abrasion with sandpaper and cleaning with isopropyl alcohol was completed to lower the skin impedance to <10 Ω (Ω). The myoelectric signals were acquired by surface EMG system (MyoMuscle, Noraxon, USA) at the sampling frequency of 1024 Hz, band width of 20 to 500 Hz and common-mode rejection ratio greater than 80 dB. Bipolar EMG electrodes were placed with 1 cm inter-electrode distance. Placement of EMG electrodes for the 6 selected muscles (bilateral IO, UGMax, LGMax, GMed and BF of the exercising leg) were accorded to the recommendation reported previously (Table 2) [15–17].

**Table 2:** EMG electrode placements

Muscle	EMG electrode placement
Internal obliques (IO)^a^	1 cm medial to the anterior superior iliac spines, *below a line connecting the left and right anterior superior iliac spines* [15]
Upper division of gluteus maximus (UGMax)	Two finger’s width above the midpoint of the line formed between the posterior superior iliac spine of the innominate and greater trochanter of the femur [16]
Lower portion of gluteus maximus (LGMax)	Immediately below the line formed by the posterior superior iliac spine of the innominate and greater trochanter of the femur [16]
Gluteus medius (GMed)	33% of the distance from iliac crest to greater trochanter, starting from greater trochanter [17]
Biceps femoris (BF)	35% of the distance from ischial tuberosity to lateral side of popliteal fossa, starting from ischial tuberosity [17]

^**a**^Activity of IO on both sides were collected

Participants initially performed voluntary contraction, crunch and reverse crunch in crook-lying position against manual resistance to their maximum effort for 3 repetitions respectively, so that EMG data of maximal voluntary isometric contraction (MVIC) of bilateral IO was collected [18]. Each repetition lasted for 5 s with 10-s rest interval. Participants were required to perform the hip exercises naturally (NCA) and followed by ECA conditions with their dominant leg. For exercise trials under the NCA condition, participants were instructed to perform the studied hip exercises naturally without any instruction or correction specified by the examiner. For trials under the ECA condition, participants were guided individually to activate the muscles of their transverse abdominal wall using the abdominal wall bracing manoeuvre described in previous studies [9, 10, 19]. Participants were instructed to perform the abdominal wall bracing manoeuvre by “bringing their navel up and in toward the spine, then tightening up the abdominal wall muscles without causing any change in the position of their lumbar spine”. Training of the ECA was performed under the guidance of the student therapist until participants were able to contract the abdominal core muscles up to 20% MVIC of their own IO without difficulty. On average, the training of ECA took approximately 15 min. During the execution of the hip exercises, the activation level of IO was verified by the real-time monitoring to differentiate the NCA and ECA conditions. Palpation of abdominal wall bracing during the ECA practice without any movement of the pelvis or lower lumbar spine was applied to ensure the proper activation of the core muscles in addition to the real time monitoring of the EMG activity recorded over the bilateral IO. The MVIC of the GMed, UGMax, LGMax and BF were determined after all exercises by standardized procedures of manual muscle testing against resistance by the assessor. For gluteus medius, the MVIC was performed with hip abduction in the side-lying position against manual resistance applied just above ankle with the hip in neutral rotation and slightly extended [20]. For gluteus maximus, MVIC was performed in the prone position with the knee flexed to 90° and the hip extended with resistance applied just above the knee [18]. The MVIC of the hamstring muscles was performed in the prone position, with the knee flexed 45° with resistance applied just above the ankle [20]. A belt was used to stabilize the respective body parts proximal to the application of manual resistance during the MVIC procedures.

Three hip exercises performed by the participants included:

1. Clam
  Participants were asked to adopt the side-lying position with their hip flexed 45° and knees flexed to 90° and to perform the clam exercise with the dominant leg (30° abduction and 30° external rotation of the hip in a combined manner).
2. Hip abduction in side-lying (HABD)
  Participants were asked to adopt the side-lying position and perform the hip abduction to 30° with the dominant leg with knee fully extended, while the non-dominant leg was flexed to provide stability.
3. Prone hip extension (PHE)
  Participants were asked to adopt the prone lying position with the knee of the dominant side flexed to 90° and perform the hip extension exercise up to 20°.

HABD was selected due to the highest activation of GMed shown in a previous study [21]. Sidorkewicz et al. [22] suggested that when minimal activation of tensor fascia latae is desired, Clam is preferred over HABD. Although GMax showed the highest activation in single-leg squat, PHE is more commonly used in rehabilitation due to its simplicity [23].

**Fig. 1:**
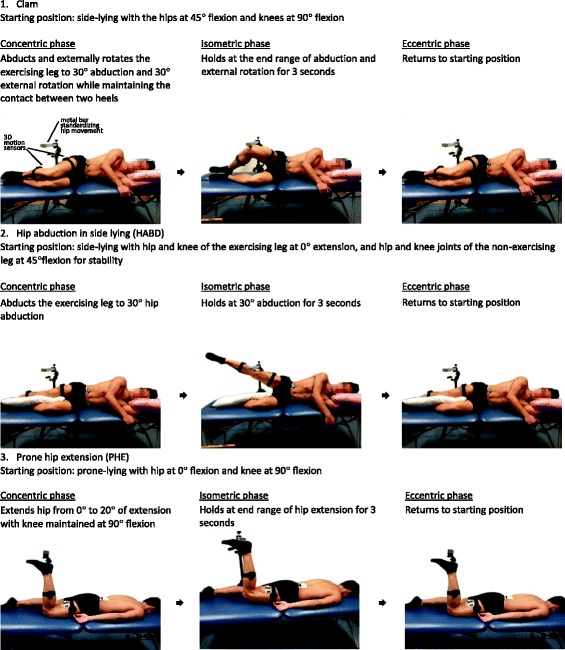
Detailed descriptions and illustrations of the three defined phases of hip exercises

The sequence of the exercises was randomized by drawing lots. All exercises were instructed by a student physiotherapist and practice trials were allowed. The pace of exercise was standardized with the metronome set at 60 beats per minute. Each exercise consisted of three trials, with each trial containing concentric, isometric and eccentric phases. Detailed descriptions and illustrations of the three corresponding phases of each exercises are presented in Fig. 1. Each phase lasted for 3 s with 3-s rest in between trials. 1-min rest was given between exercises.

The joint angles of the lumbar spine, hip and knee joints were monitored and standardized within participants between trials using the 3D motion analysis system (MyoMotion system, Noraxon, USA). Four motion sensors would be placed as follows: 1) spinous process of L1; 2) S2; 3) lateral side of middle thigh and 4) middle of the fibula of the dominant leg in order to affirm the 3 phases defined in each exercise. A metal bar was set as a target for the participants in order to standardize end range of movement in three exercises with reference to the hip angle (flexion and abduction for Clam exercise, abduction for HABD exercise, and extension for PHE exercise).

### Data processing and statistical analysis

Raw EMG signals were filtered using 4th order Butterworth filter (band-pass of 10–500 Hz) and root mean square (RMS) of the moving window of 50 ms. Maximal EMG of every muscle was obtained in the peak 1-s moving average from the whole MVIC trial. The %MVIC is then computed by normalizing the RMS value to the MVIC value. Mean %MVIC of all muscles of 3 trials were calculated. Invalid trials (%MVIC of either IO below or above 20% in ECA and NCA conditions respectively) were identified and replaced by the series mean of %MVIC of respective muscles in that particular exercise, phase and condition, for analysis (Table 3).

**Table 3:** Number of participants with invalid trials

Exercise	Condition	Phase	Number of subjects with invalid trials (*n*/20)
Clam	ECA	Concentric	2
	ECA	Eccentric	6
HABD	NCA	Concentric	1
	NCA	Isometric	4
	ECA	Concentric	2
	ECA	Eccentric	5
PHE	NCA	Isometric	1
	ECA	Eccentric	4

*HABD* Hip abduction, *PHE* Prone hip extension, *ECA* Enhanced core activation, *NCA* Natural core activation

Statistical analysis was performed using IBM SPSS Version 23.0 (IBM SPSS, Inc., Armonk, NY). Test-retest reliability of the EMG data recorded in 3 trials was determined by Intra-class Correlation Coefficient (ICC 3,1). Normality of EMG data distribution was tested with the Shapiro-Wilk test. Separate analysis of paired t-tests or Wilcoxon signed-rank tests was used initially to determine differences in muscle recruitment between two core conditions and differences in activation between UGMax and LGMax. Differences in %MVIC between NCA and ECA in each exercises and phases were calculated and the difference between exercises within phases are examined using One-way Repeated Measures Analysis of Variance (ANOVA) or Friedman test. Post hoc analysis with Bonferroni correction was used in the cases of significant main effects. In addition, the association between selected characteristics of the participants (gender, physical activity level and TMG data) and the difference in %MVIC between 2 core conditions are examined with Spearman’s rho. Based on the suggestion of a reviewer, analysis of covariance (ANCOVA) was conducted for factors with significant correlation to review the effects of covariates. The level of significance for all statistical analysis was established at 0.05.

## Results

### Reliability of EMG Recordings

The test-retest ICCs (3,1) for the EMG recordings of the hip muscles during Clam, HABD and PHE in concentric, isometric and eccentric phases were summarized in ranges. Good to excellent reliability [24] was observed in Clam in NCA (0.698–0.977) and ECA (0.681–0.967). Similarly, good to excellent reliability was observed in HABD in NCA (0.700–0.983) and ECA (0.596–0.964). Fair to excellent reliability was observed in PHE in NCA (0.440–0.968) and ECA (0.669 to 0.922).

### Emg activity level during the hip exercises

#### Threshold of the abdominal core activation

The activities of bilateral IO in all phases of all exercises were significantly higher in ECA condition than that in NCA condition (Table 4).

**Table 4:** EMG activity in terms of %MVIC with mean (SD) of internal oblique muscle over the contralateral side (CIO) and ipsilateral side (IIO) of the exercising hip

Exercise	Phase	CIO			IIO		
		NCA	ECA	*P*-value	NCA	ECA	*P-*value
Clam	Concentric	2.45 (0.51)	47.08 (4.98)	<0.001^b^	4.58 (0.81)	40.75 (3.92)	<0.001^b^
	Isometric	2.49 (0.50)	40.05 (4.18)	<0.001^b^	6.95 (1.18)	42.88 (4.40)	<0.001^b^
	Eccentric	2.26 (0.49)	37.82 (3.14)	<0.001^b^	3.61 (0.60)	38.81 (3.26)	<0.001^b^
HABD	Concentric	3.05 (0.59)	43.03 (3.67)	<0.001^b^	11.12 (1.08)	73.99 (6.56)	<0.001^a, b^
	Isometric	2.48 (0.33)	41.71 (3.35)	<0.001^b^	11.32 (0.94)	65.68 (6.48)	<0.001^a, b^
	Eccentric	2.69 (0.52)	37.09 (2.95)	<0.001^b^	7.96 (0.94)	54.87 (4.40)	<0.001^a, b^
PHE	Concentric	6.14 (0.91)	60.94 (6.22)	<0.001^a, b^	4.14 (0.73)	58.34 (6.58)	<0.001^b^
	Isometric	7.98 (1.12)	63.12 (7.37)	<0.001^b^	5.51 (0.86)	46.33 (3.74)	<0.001^b^
	Eccentric	5.84 (0.99)	40.60 (3.62)	<0.001^b^	3.60 (0.64)	36.81 (2.74)	<0.001^b^

*ECA* Enhanced core activation, *HABD* Hip abduction, *NCA* Natural core activation, *PHE* Prone hip extension

^**a**^paired t-test otherwise Wilcoxon Signed rank test, ^**b**^indicates significance

#### Activation of hip muscles in NCA vs. ECA conditions

**Fig. 2:**
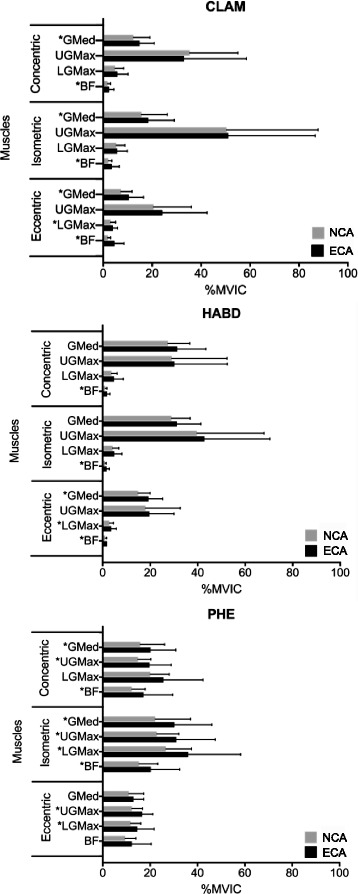
Comparisons of EMG activity of hip muscles in terms of %MVIC with mean and SD during the three hip exercises performed between NCA and ECA conditions (* indicates *P* value < 0.05)

Table 5 and Fig. 2 show the comparisons of the activation of 4 hip muscles between NCA and ECA condition in 3 hip exercises. GMed demonstrated significantly higher activation (*P* < 0.05) in ECA than in NCA condition in all phases of Clam. However, it only showed significantly greater activations (*P* = 0.004) under ECA condition in the eccentric phase of HABD. Particularly in PHE, the activity of GMed was greater in ECA condition in concentric (*P* = 0.002) and isometric (*P* < 0.001) phases. UGMax showed significantly greater activations (*P* < 0.05) in ECA condition in all phases of the PHE, whereas there was no significant difference in the activation level of the UGMax in all phases of Clam and HABD (*P* > 0.05). In LGMax, activation in ECA condition was significantly higher than that in NCA condition in the eccentric phase of all 3 exercises (*P* < 0.05), and in the isometric phase of PHE (*P* = 0.013). BF showed significantly greater activations (*P* < 0.05) in ECA condition when compared to that in NCA condition in all phases of the 3 hip exercises except in the eccentric phase of PHE (*P* = 0.074).

**Table 5:** Comparisons of EMG activity in terms of %MVIC with mean (SD) of the hip and thigh muscles between two core pre-activation conditions

Exercise	Phase	GMed			UGMax			LGMax			BF		
		NCA	ECA	*P-*value	NCA	ECA	*P*-value	NCA	ECA	*P-* value	NCA	ECA	*P-*value
Clam	Concentric	12.27 (6.79)	14.89 (5.93)	0.026^a, b^	35.37 (19.68)	33.01 (25.50)	0.405	4.90 (3.42)	5.81 (4.33)	0.071	1.75 (1.33)	2.41 (2.00)	0.008^b^
	Isometric	15.63 (10.53)	18.39 (10.66)	0.002^b^	50.41 (37.42)	51.07 (35.56)	0.796	5.28 (3.60)	5.71 (4.16)	0.219	2.12 (1.42)	3.44 (3.18)	0.033^b^
	Eccentric	7.27 (4.48)	10.48 (5.92)	0.007^b^	20.50 (15.52)	24.12 (18.38)	0.088	3.04 (2.03)	3.94 (1.93)	0.003^b^	1.87 (1.24)	4.64 (3.83)	0.001^b^
HABD	Concentric	27.52 (9.1)2	31.38 (12.02)	0.057^a^	29.06 (23.31)	30.20 (22.30)	0.728	3.75 (2.38)	4.80 (3.85)	0.058	1.08 (0.72)	1.86 (1.20)	0.005^b^
	Isometric	28.89 (7.92)	31.20 (10.10)	0.096	39.63 (28.29)	42.91 (27.47)	0.466	4.19 (2.67)	4.88 (3.24)	0.058	0.98 (0.55)	1.70 (1.05)	0.003^b^
	Eccentric	14.89 (5.02)	19.33 (5.94)	0.004^a, b^	17.97 (14.69)	19.56 (10.51)	0.353	2.79 (1.76)	3.63 (2.11)	0.004^b^	1.03 (0.68)	1.88 (0.86)	<0.001^b^
PHE	Concentric	15.81 (10.28)	20.24 (10.57)	0.002^b^	14.82 (5.47)	19.74 (9.12)	0.002^a, b^	20.09 (8.00)	25.62 (16.68)	0.071	12.26 (5.66)	17.23 (12.28)	0.019^b^
	Isometric	22.15 (14.90)	30.28 (15.84)	<0.001^b^	22.87 (9.21)	30.98 (16.51)	0.004^b^	26.62 (10.82)	36.01 (22.27)	0.013^b^	15.27 (7.91)	20.30 (12.21)	0.008^b^
	Eccentric	10.97 (6.32)	12.92 (4.40)	0.121	12.32 (4.43)	16.62 (4.46)	<0.001^a, b^	11.73 (4.27)	14.56 (7.12)	0.021^b^	9.47 (4.50)	12.25 (8.20)	0.074

*BF* Biceps femoris, *ECA* Enhanced core activation, *HABD* Hip abduction, *GMed* Gluteus medius, *LGMax* Lower part of gluteus maximus, *NCA* Natural core activation, *PHE* Prone hip extension, *UGMax* Upper part of gluteus maximus. ^a^indicates paired t-test otherwise Wilcoxon Signed rank test was used and ^b^indicates significant difference between two core conditions

#### Difference in the activation of UGMax & LGMax

In Clam and HABD, the %MVIC of UGMax was significantly greater than that of LGMax in all phases for both ECA and NCA conditions (*P* < 0.01). In PHE, there were no significant difference between %MVIC of UGMax and that of LGMax in most phases for both NCA and ECA conditions, except in the concentric phase of the PHE under NCA condition, the %MVIC of LGMax was significantly greater than that of UGMax (*P* = 0.003) (Table 6).

**Table 6:** Comparisons of the difference in UGMax and LGMax activity of in terms of %MVIC with mean (SD) of the hip and thigh muscles between two core pre-activation conditions

		%MVIC difference (UGMax – LGMax)			
Exercise	Phase	ECA		NCA	
		Mean difference	*P-*value	Mean difference	*P*-value
Clam	Concentric	27.19 (18.29)	0.001^b^	30.47 (21.12)	<0.001^b^
	Isometric	45.36 (25.32)	<0.001^b^	45.13 (26.58)	<0.001^b^
	Eccentric	20.18 (13.07)	0.001^b^	17.46 (11.07)	<0.001^b^
HABD	Concentric	25.40 (16.00)	<0.001^b^	25.31 (16.57)	<0.001^b^
	Isometric	38.03 (19.56)	<0.001^b^	35.45 (20.09)	<0.001^b^
	Eccentric	15.93 (7.58)	0.001^b^	15.18 (10.46)	<0.001^b^
PHE	Concentric	−5.87 (13.44)	0.057	−5.27 (6.81)	0.003^a b^
	Isometric	−5.03 (19.60)	0.086	−3.74 (8.10)	0.061^a^
	Eccentric	2.07 (5.94)	0.121	0.59 (3.60)	0.477^a^

*ECA* enhanced core activation, *HABD* Hip abduction, *LGMax* Lower part of gluteus maximus, *NCA* Natural core activation, *PHE* Prone hip extension, *UGMax* Upper part of gluteus maximus

^a^indicates paired t-test otherwise Wilcoxon Signed rank test was used and ^b^indicates significant difference between two core conditions

#### Difference in change of activation of hip muscles between exercises

The change of EMG activities of GMed in isometric phase (F = 5.900, *P* = 0.005), BF in concentric (*P* = 0.024) and isometric phases (*P* = 0.017) after enhanced core activation significantly differed between the three hip exercises. With respect to GMed, post hoc analysis revealed that enhancement of EMG activity in PHE was significantly greater than that in HABD (*P* = 0.009). There was no statistical difference for the comparisons between PHE and Clam, HABD and Clam. With respect to BF, post hoc analysis did not reveal any significant difference among exercises.

### Correlation analysis

Radial displacement (Dm) of LGMax measured by TMG had significant correlation with enhancement of LGMax activity level in the concentric (*r* = −0.514, *P* = 0.020) and eccentric phases (*r* = −0.519, *P* = 0.019) of HABD. In addition, physical activity level of participants had positive correlation with the enhancement of GMed (*r* = 0.511, *P* = 0.021) and UGMax (*r* = 0.548, *P* = 0.012) activities in the eccentric phase of Clam. However, gender and contraction time (Tc) measured by TMG did not show significant correlation with the change of EMG activity of hip muscles in all phases of the hip exercises.

## Discussion

The purpose of this study was to examine if abdominal core activation could affect the recruitment of the hip muscles and the extent of difference across various phases and exercises. Furthermore, factors such as gender, physical activity level and the contractile properties of GMax were investigated whether it would impose confounding effect on the activation of the hip muscles. Our results demonstrate that abdominal core activation could enhance the recruitment of hip muscles to a small to moderate extent during various phases of the therapeutic hip exercises under examination.

One of the possible explanations for the general trend of the enhanced activity of hip muscles under the ECA condition could be explained by the functional anatomy and biomechanics of the lumbo-pelvic-hip complex. The GMed, GMax and BF all have the muscle origin and attachment at various parts of the innominate bone and the femur respectively. Under the circumstance when enhanced recruitment of the abdominal core muscles is achieved while hip exercises were performed under the ECA condition, stability of the lumbar spine and pelvis would have been augmented [25]. As a result, compensatory movements occurring in the lumbar spine and pelvis during the hip exercises can be minimized. Oh et al. [5] found that abdominal drawing-in maneuver decreased lumbar hyper-extension, excessive anterior pelvic tilt and reduced erector spinae activity during PHE. Meanwhile in side-lying hip abduction, core activation [6] or external stabilization methods [26] could reduce the lateral pelvic tilt and quadratus lumborum activity. These findings indicated that the unwanted pelvic movements, which might contribute to the composite movement during hip exercises, would be minimized with abdominal core activation. Therefore, relatively higher amplitude of EMG signals of the prime hip muscles would be required to achieve the same range of movements under abdominal core activation, i.e. enhanced activity of UGMax, LGMax and BF in PHE; enhanced activity of UGMax, GMed in Clam and HABD. Enhancement of muscles activity in abdominal core activation conditions was not only evident for the prime hip muscles, but also for the non-prime movers. For instance, there was higher GMed activity in PHE after core activation. This could be explained that GMed involves three functional portions, with the posterior portion contributing to hip extension [27].

Differences in activation of the UGMax and LGMax were evident in the hip exercises. UGMax was preferentially recruited in Clam and HABD while UGMax and LGMax were equally activated in the isometric and eccentric phases of PHE. Our findings concur the recruitment pattern of GMax reported by Selkowitz et al. [8] and Lyons et al. [28] in which the UGMax is predominantly functioning as the hip abductor and external rotator, whereas the LGMax mainly acts as a hip extensor. With reference to the fiber orientation of the UGMax in which it inserts into the fascia latae and hence it facilitates hip abduction, while LGMax contains a larger moment arm for hip extension [29]. Our finding supports the concept that the motor units throughout a muscle are not uniformly distributed. Compartments of a muscle could be functionally differentiated, which implies that the portions of muscle should be taken into consideration while prescribing exercises for patients with specific pathological conditions. For instance, exercise targeting UGMax may be prescribed for correcting hip excessive adduction and internal rotation. The strength of GMax may also be tested in different positions targeting specific portions, instead of a single testing position for testing GMax as a hip extensor only.

Although the activation of abdominal core muscles could enhance the hip muscle activities in the three hip exercises, the extent of the net increase in EMG activity with ECA compared to NCA was relatively small. It remains difficult to postulate the clinical significance for its degree of promotion of activation of the prime hip muscles during dynamic hip exercises with the ECA trials. Future study is recommended so as to evaluate if variations in responses towards the enhanced abdominal core activity during active hip exercises would be displayed in individuals with either hip or lumbar spine dysfunction. Furthermore, the magnitude of enhancement did not differ significantly between exercises. As mentioned previously, abdominal core activation reduced lumbo-pelvic movements in different directions during hip exercises. It is postulated that abdominal core activation stabilizes the lumbo-pelvic region to a similar extent for different hip movements. Therefore, the promotion of hip muscles activity in enhanced core condition is independent of the type of hip exercises. However, due to the large variance of the changes in the hip muscle activity under the ECA trials, caution is recommended when interpreting the results of the present study. There are some limitations with regards to the electrode placement and cross-talk of the myoelectric signals when using surface EMG method to quantify the level of muscle activity of the gluteal region. For the EMG electrode placement of GMed and BF adopted in this study, the possible influence related to the variation of EMG activity at and near the innervation zone of the respective muscle should be acknowledged. It is also important to take into consideration of the possibility and issues of cross-talk of the myoelectric activity from the nearby muscle(s) when using the surface EMG method. For the EMG electrode placement for GMed adopted in this study, the more distal the electrodes were placed along the line formed between the iliac crest of the innominate bone and the greater trochanter of the femur, the greater the likelihood of cross-talk from the GMax we would have. To overcome this limitation, further study using the intramuscular EMG approach is recommended to examine the myoelectric activity of the individual gluteal muscles and the respective subdivisions of the same gluteal muscle.

Only the physical activity level and radial displacement of LGMax showed significant correlation with the change of specific hip muscles activity in specific phases and exercises. The results did not suggest that the factors being investigated imposed a major confounding effect on the hip muscles activity level in response to abdominal core activation. Therefore, ANCOVA was not performed in the present study. Interestingly, the radial displacement of LGMax negatively correlated with the change of LGMax activities. García-Manso et al. [29, 30] stated that low values of Dm demonstrate high level of muscle stiffness. The mechanism of muscle stiffness affecting the muscle recruitment specifically in abdominal core activation is yet to be established.

Regarding the MVIC procedure adopted in our study, 2 portions of the GMax were tested using resisted isometric contraction in hip extension. This position might not be able to obtain the true MVIC of UGMax because UGMax functions as a combination of hip extensor, abductor and external rotator instead of an extensor only. However, since the comparison of the muscle activities was conducted using %MVIC, the difference found in UGMax between the two abdominal core conditions remain valid. This could be improved by having the muscle strength test of the UGMax in different testing positions in the future study. Another limitation of this study is the limited sample size, which would lower the statistical power. In addition, characteristics of the participants are very similar, for example they are all young age and have moderate to high activity levels. This may limit the generalizability of our study and make it less comparable to all populations. However, we believe that the findings are applicable to the population with similar characteristics as our recruited participants. Further study with larger sample size and inclusion of other populations, for example the elderly and patients with various hip pathologies, would be needed to find that if the results would be similar cross populations.

For the analysis of the data, if the %MVIC of IO of both sides did not fulfill the condition defined as ECA and NCA with 20%MVIC as thresholds, the data of all muscles at that specific phase was considered as invalid. The invalid data were replaced by mean value of all other %MVIC of corresponding muscle in the population at the specific phase. According to Little and Rubin [31], the mean substitution approach might lead to some statistical pitfalls, including overestimation of sample size, underestimation of variance, negative bias on correlation and distortion of shape of distribution. Since the number of invalid trials identified from the respective phases of the three hip exercises was relatively low (Table 3), it is unlikely that the aforementioned issues would have a substantial effect on the present findings though interpretation with care may apply.

## Conclusion

Abdominal core activation using the abdominal wall bracing manoeuvre showed a small to moderate level of positive effect on promoting the hip muscles recruitment in the therapeutic exercises Clam, HABD and PHE (with the increase of 0.7% to 9.4% of MVIC). Compensatory movements such as pelvic tilt and hyperextension of the lumbar spine were suggested to be reduced by stabilizing the pelvis with abdominal core activation. The present findings suggest the potential benefit of abdominal core activation in enhancing the strengthening effect of exercises for rehabilitation of the lower limbs. However, the clinical significance of using enhanced abdominal core activation in promoting the effect extent of therapeutic hip exercise is yet to be examined due to the small to moderate level of positive effect found in this study. In addition to enhancement of hip activation in ECA condition, more enhancement in hip activation is correlated to higher physical activity level and stiffer muscle in this study. Further research in patient groups with either hip pathology or other lower limb conditions using the prospective study design is recommended to evaluate the effectiveness of therapeutic hip exercises with abdominal core activation, and to study the confounding effect of physical activity and contractile property of muscle on hip activation.

## Abbreviations

ANCOVA: Analysis of covariance
ANOVA: Analysis of variance
BF: Biceps femoris
CIO: Contralateral side internal oblique
Dm: Maximal radial displacement
ECA: Enhanced core activation
GMax: Gluteus maximus
GMed: Gluteus medius
HABD: Hip abduction exercise
ICC: Intra-class correlation coefficient
IIO: Ipsilateral side internal oblique
IO: Internal oblique
IPAQ-SF: International physical activity questionnaire short form
LGMax: Lower part of gluteus maximus
MVIC: Maximal voluntary isometric contraction
NCA: Natural core activation
PBU: Pressure biofeedback unit
PHE: Prone hip exercise
Tc: Contraction time
TMG: Tensiomyography
UGMax: Upper part of gluteus maximus
